# Supplementary material for: Specific residues in the cytoplasmic domain modulate photocurrent kinetics of channelrhodopsin from Klebsormidium nitens
Source: Commun Biol. 2021 Feb 23;4:235. doi: 10.1038/s42003-021-01755-5 (PMC7902849; doi:10.1038/s42003-021-01755-5)
Supplement: Supplementary file 2 — Supplementary information [file 42003_2021_1755_MOESM2_ESM.pdf]

Supplementary information

Specific residues in the cytoplasmic domain modulate photocurrent kinetics of channelrhodopsin from *Klebsormidium nitens*

Authors
Rintaro Tashiro[1], Kumari Sushmita[2], Shoko Hososhima[1], Sunita Sharma[2], Suneel Kateriya[2], Hideki Kandori[1], Satoshi P. Tsunoda[1]

Affiliations
Department of Life Science and Applied Chemistry, Nagoya Institute of Technology, Showa-ku, Nagoya 466-8555, Japan.
Laboratory of Optobiology, School of Biotechnology, Jawaharlal Nehru University, New Delhi, 110067, India

Corresponding author
Satoshi Tsunoda email: tsunoda.satoshi@nitech.ac.jp

- Supplementary Figures 1-9
- Supplementary Tables 1-4

```
                                                                  <----
BR         1  -----------------------------------------------------QAQITGR   7
CrChR2     1  --MDYGGALSAVGRELLFVTNPVVVNG-SVLVPEDQCYCAGWIESRGT---NGAQTASNV  54
PsChR      1  ---------------MGFQLNPEYLNE-TILLDD----CTPIYLNVGP---LWEQKVARG  37
KnChR      1  MTVDAHSTVDA--------HSTVTDSH-SAGNGTESCYVADFLGMHHESHEGALYSVYKS  51
GtCCR4     1  ----------------------MTTSAPSLSDPNWQYGMGGWNNPRLPNFNL-HDPTVIG  37

              ------TM1------>                <-----------TM2---------->
BR         8  PEWIWLALGTALMGLGTLYFLVKGMGVSDPDAKKFYAITTLVP-------AIAFTMYLSM  60
CrChR2    55  LQWLAAG-----FSILLLMFYAYQTWKSTCGWEEIYVCAIEMV-------KVILEFFFEF 102
PsChR     38  TQWFGVI-----LSLAFLIYYIWITYKATCGWEELYVCTIEFC-------KIVIELYFEF  85
KnChR     52  LEWGCFL-----ISIGLFVFYLQQYRKKTAGWEVIYIAFIESF-------KYIFEIFWPH  99
GtCCR4    38  VDWLGFL---CLLGASLALMYKLMSFKGPDGDQEFFVGYREEKCLSIYVNLIAAITYWGR  94
               :*         :.                .  : ::                    :

                  <---------TM3-------->            <----TM4-----
BR        61  LLGYGLTMVPFGGEQNPIYWARYADWLFTTPLLLLDLALLVDADQG---TILALVGADGI 117
CrChR2   103  ---KNPSMLYL-ATGHRVQWLRYAEWLLTCPVILIHLSNLTGLSNDYSRRTMGLLVSDIG 158
PsChR     86  ---SPPAMIYQ-TNGEVTPWLRYAEWLLTCPVILIHLSNITGLNDDYSGRTMSLITSDLG 141
KnChR    100  ---NNPAQLNIYGVNKSVPWVRYMEWMITCPVILMALSNISGEEGEYTHRSMQLLATDQG 156
GtCCR4    95  IC---AHFNNDMGLSLSVNYFKYLDYIFTCPILTLDLLWSLNLPYKITYSLFV-----GL 146
                                 : :* ::::* *:: : *    .         :

              -------->       <-----------TM5------------>     <-----
BR       118  MIGTGLVGALTKVYSYRFVWWAISTAAMLYILYVLFFGFTSKAESM--RP--EVASTFKV 173
CrChR2   159  TIVWGATSAM----ATGYVKVIFFCLGLCYGANTFFHAAKAYIEGYHTVPKGRCRQVVTG 214
PsChR    142  GICMAVTSAL----SKGWLKWLFFVIGCCYGASTFYHAALIYIESYYTMPHGVCKNMVLA 197
KnChR    157  AILCAITAAA----SEGAISAVFYAIGVCYGICTFYFCLQIYIEAYFTLPE-TCHSAVKW 211
GtCCR4   147  TIACGVFCNAF-EPPARYLWFMFGCFIFAFTWISIIRLVYARFQQFLNEDAKKIRAPLKL 205
               *  .             :   :      :    :        :             .

              ------TM6------->         <-----------TM7------------>
BR       174  LRNVTVVLWSAYPVVWLIGSEGAGIVPLNIETLLFMVLDVSAKVGFGLILLRSRAIFGEA 233
CrChR2   215  MAWLFFVSWGMFPILFILGPEGFGVLSVYGSTVGHTIIDLMSKNCWGLLGHYLRVLIHEH 274
PsChR    198  MAAVFFTSWFMFPGLFLAGPEGTNALSWAGSTIGHTVADLLSKNAWGMIGHFLRLEIHKH 257
KnChR    212  MAVIFYAGWLCYPCFFLAGSEGWGNLSYEGSAIGHCIADLLSKNAWGVMHWWIRCQLEEY 271
GtCCR4   206  SLTLYFSIWCGYPALWLLTE--FGAISQLAAHVMTVIMDVAAKSVYGFALLKFQLGVDKR 263
                 :    *  :*.::       . :      :   :  *:  :*  :*.     :  . :

BR       234  EAP--------------EP----SAGDGAAATSD-------------------------- 249
CrChR2   275  ILIHG--------D---------------------------------------------- 280
PsChR    258  IIIHG--------DVRRPI----TVNT---------------LGREVTVSCFVDKE-EED 289
KnChR    272  KHTHN--------GQLPHY----SLETRAKMRAEAGHIIAGSLGSVLHVAGHQH------ 313
GtCCR4   264  DVWLDELKSVRYRDVVPQIRPSKTRESR-MEYSEDGDFMRPSKGKRAEGDYMNPRWDHHD 322

BR       250  ------------------------------------------------------------ 249
CrChR2   281  ------------------------------------------------------------ 280
PsChR    290  EDERISTKTYANRASFMKMRNDMEQRGIQTRKS-LE----MLAPPPALNDGSIV-LA--- 340
KnChR    314  --------------HHFHFDEDSGMQGAQIQGQPLAQANERANLKASLLNGNAVNYNQAV 359
GtCCR4   323  DGRRLPDS--------REMDEQVHEKDQEIS-STMKQIADLNKQLSAMQESEAV------ 367

                                                                  <----
BR       250  ------------------------------------------------------------ 249
CrChR2   281  ------------------------------------------------------------ 280
PsChR    341  VADPMTLTFFTQQLSQ-LDATIRATPAMGQGQLEQVLEKGGFDGVLVSPEYIQQVGLVQ- 398
KnChR    360  QAQPQAFQIQSGEDGYVISMNQGESSASGSRRTTQNIEGP--KKDPVASAFSKALGSMKS 417
GtCCR4   368  ------------------------------------------------------------ 367

              ------------------------------------------------------------
BR       250  ------------------------------------------------------------ 249
CrChR2   281  ------------------------------------------------------------ 280
PsChR    399  RLKDKYHMPVYAFGWGKSSPWRSVIEGSGVDGWLEGPYFGSTFDTDALSDAIAEMQRIKT 458
KnChR    418  KVTEHFTKDLPPS-ARPDSPSRRGSARGGADAPTAQPQQSMKIDLRD----LANNPQLQQ 472
GtCCR4   368  ------------------------------------------------------------ 367

              ----------------------------FimV----------------------------
BR       250  ------------------------------------------------------------ 249
CrChR2   281  ------------------------------------------------------------ 280
PsChR    459  SYSMVVNGVGMNGAGMN----GVGMN-----GMGMDGVGMNGAGMNGVGMNGMGGYGSVG 509
KnChR    473  QLSMMLKGQMAGGYALKTDLKDLANNPQLQQQLSMMLKGQMAGNSNTN------QGSNN 526
GtCCR4   368  ------------------------------------------------------------ 367

              ------------------------------------------------------------
BR       250  ------------------------------------------------------------ 249
CrChR2   281  ------------------------------------------------------------ 280
PsChR    510  NNMHSMPMMSQ---QAVM-MPQSA--PQMMGGMSQTQQPAMMGGMQGASPHYSVGNLQNM 563
KnChR    527  GSPRARPVSEELAQPARLQKAQSARATSNLDDEIEEQAPAQMSRRPSA----RVPRAT-- 580
GtCCR4   368  ------------------------------------------------------------ 367

              ------------------------------------------------------------
BR       250  ------------------------------------------------------------ 249
CrChR2   281  ------------------------------------------------------------ 280
PsChR    564  EGQQAVGSPQVLASSWQQSALHGGMGQQQQYGQVQQMPMMVGMQTPAS---PGGVQTPPH 620
KnChR    581  ---AAADEEQAPARTLSRKISRGDGGEPVSEERLQKRPSYRAAKAAAEAADEDMTDAPAP 637
GtCCR4   368  ------------------------------------------------------------ 367

              ------------------------------------------------->
BR       250  ------------------------------------------------------------ 249
CrChR2   281  ------------------------------------------------------------ 280
PsChR    621  TMAGQPQMSPQQLQQQLYFMQQLQQRQLQQ------------------------------ 650
KnChR    638  ALARRPSARAPAADADE-ETAPVRTASLRRAGSKSPGNSLPSSPSRRVSRANSLRAPVSA 696
GtCCR4   368  ------------------------------------------------------------ 367

BR       250  ------------------------------------------------------------ 249
CrChR2   281  ------------------------------------------------------------ 280
PsChR    651  ----QQYQGGTGQR---------------------------------------------- 660
KnChR    697  SVLLCDADGDMGDFFLDQFAAVPNSKCSVKVVATPDELIDALATGRAYDFVMVPKGVITS 756
GtCCR4   368  ------------------------------------------------------------ 367

BR       250  ------------------------------------------------------------ 249
CrChR2   281  ------------------------------------------------------------ 280
PsChR    661  ------------------------------------------------------------ 660
KnChR    757  DPEISEEIRAHAQTPLVAFGPALEGMAVAEVKAHMFELDVDDYIGLPKRGQEYPVEDLEA 816
GtCCR4   368  ------------------------------------------------------------ 367

BR       250  ---------------                                              249
CrChR2   281  ---------------                                              280
PsChR    661  ---------------                                              660
KnChR    817  LVWKYKKVADDSNKD                                              831
GtCCR4   368  ---------------                                              367
```

Supplementary Figure 1. Full amino acid alignments of bacteriorhodopsin (BR), CrChR2, KnChR and GtCCR4

The transmembrane region was shown based on the membrane topology of CrChR2. Putative FimV domain in KnChR was indicated.

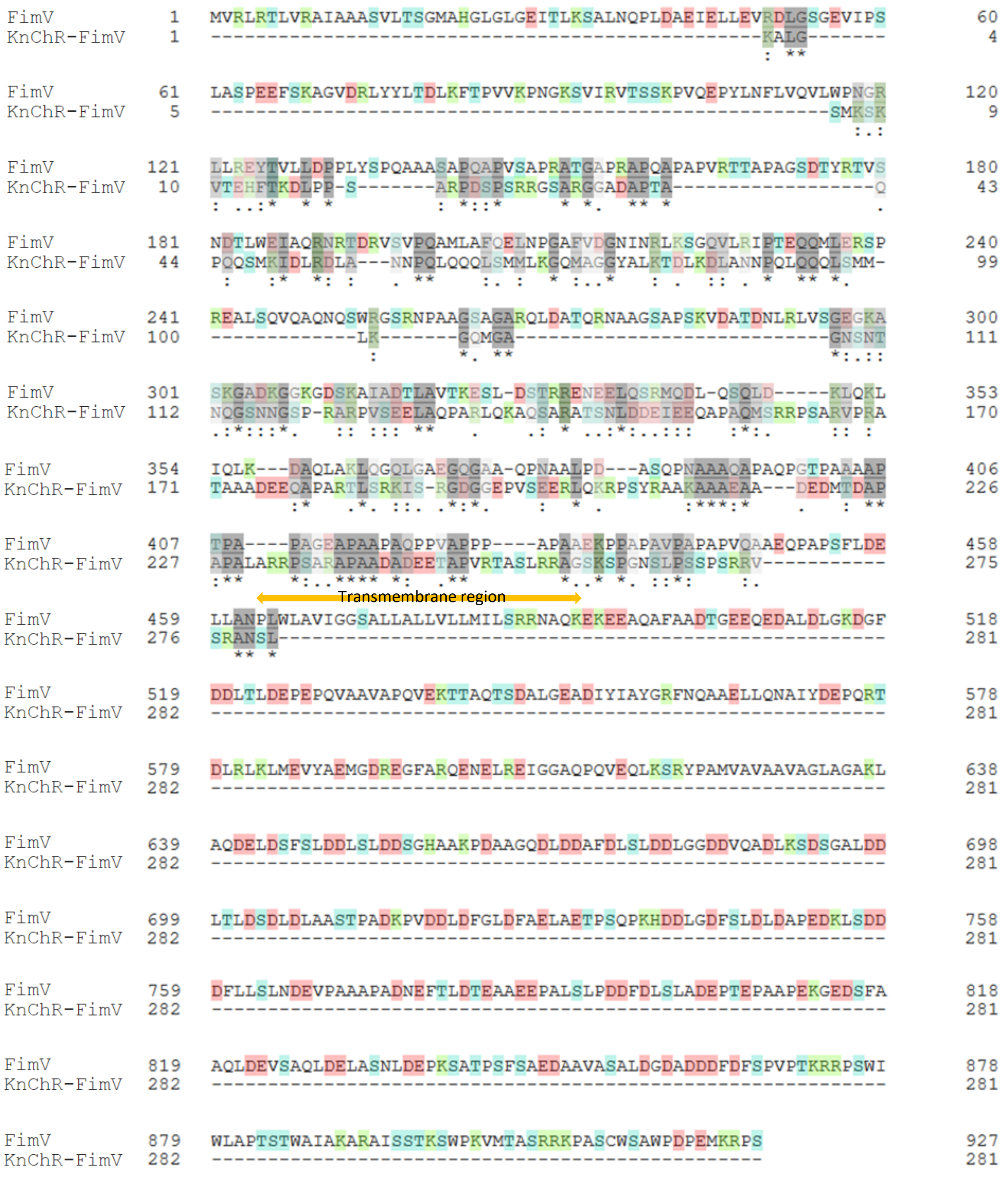

Supplementary Figure 2. Full amino acid alignments of FimV from *Pseudomonas aeruginosa* and KnChR Transmembrane region is indicated.

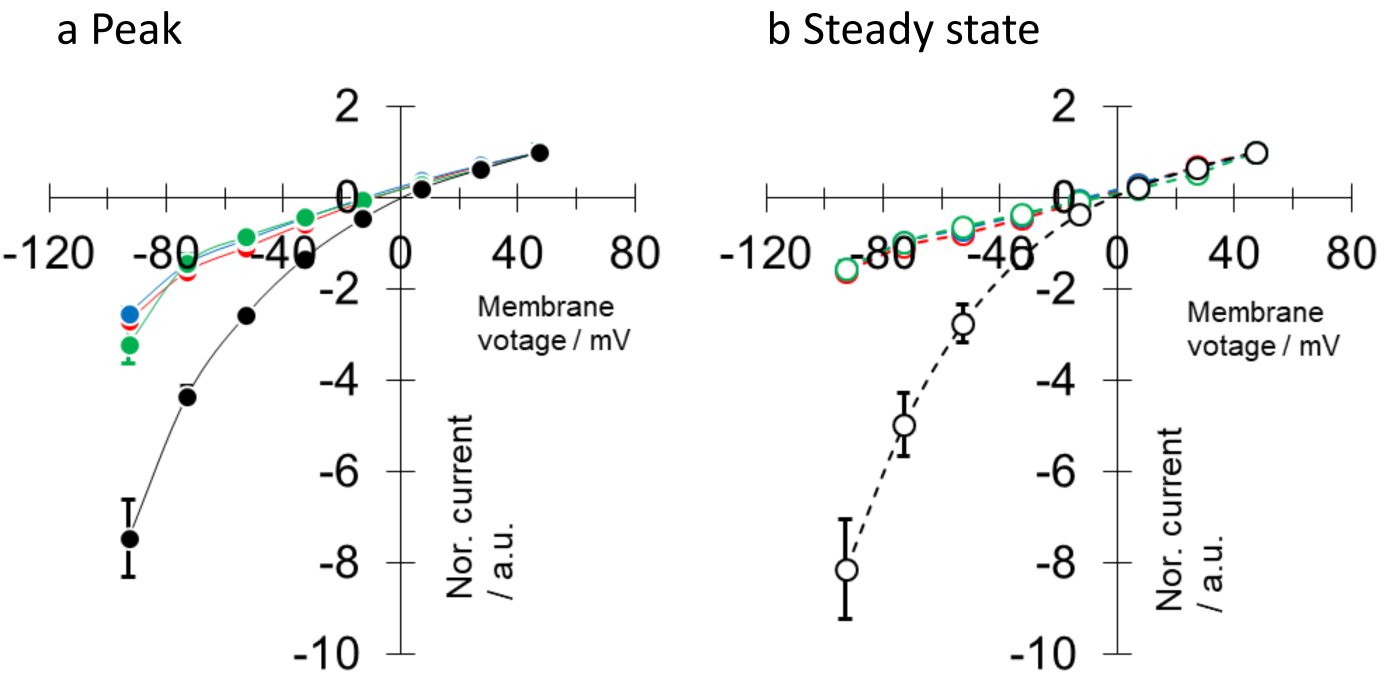

Supplementary Figure 3. I-V relationship of KnChR and CrChR2.
Photocurrent amplitude form each variant and CrChR2 is normalized (at +50 mV as 1.0). Filled (a) and empty (b) symbols indicate peak (I_p) and steady-current (I_s), respectively. Red: KnChR-272 (*N*=7), Blue: KnChR-290 (*N*=7), Green: KnChR-397 (*N*=9), Black: CrChR2 (*N*=6). Standard solutions were used (See *Materials and Methods*). Data were presented as the mean ± SEM.

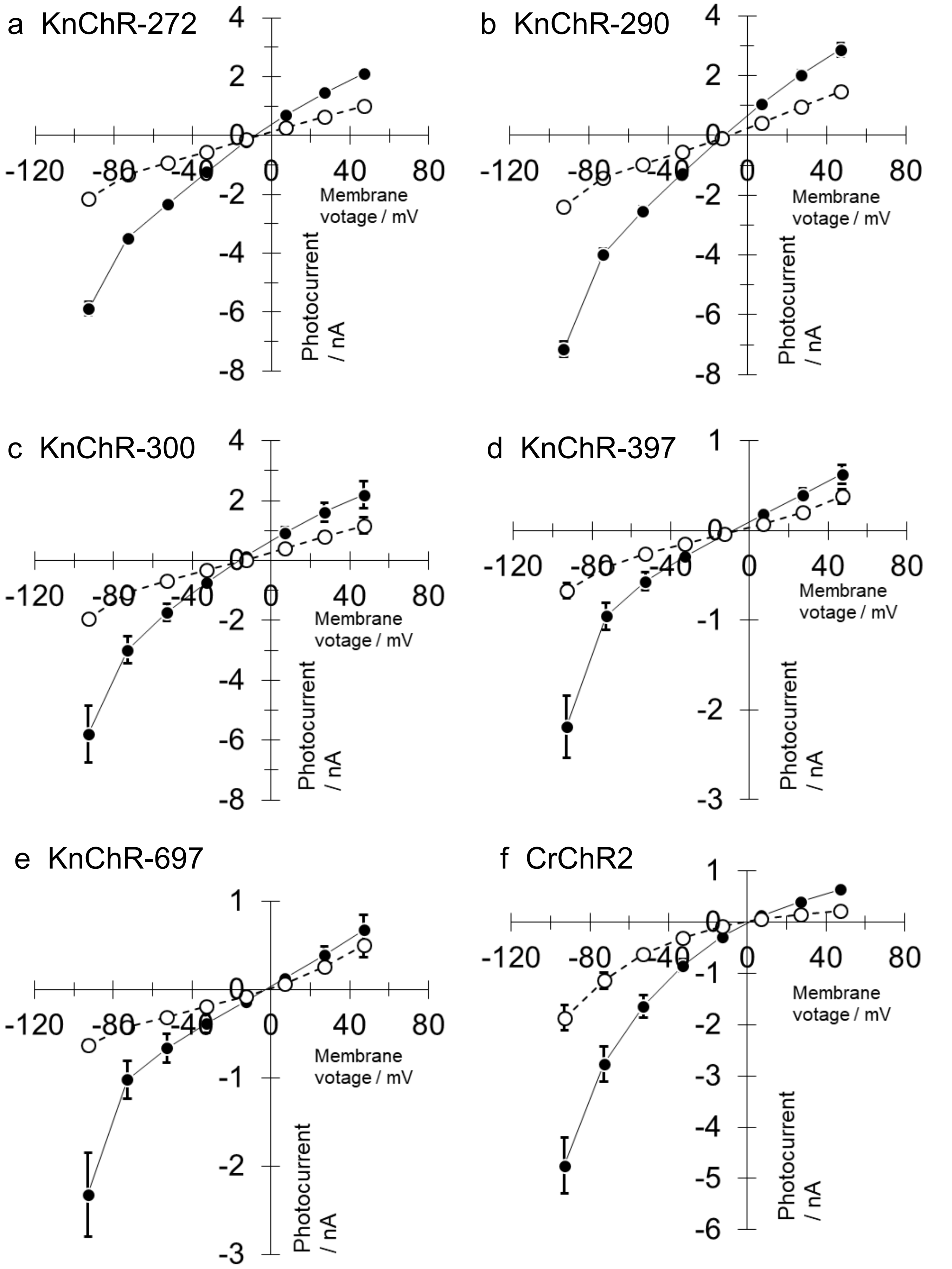

Supplementary Figure 4. I-V relationship of C-terminus length variants of KnChR and CrChR2. Amino acid length of KnChR variants is indicated on each panel (**a-e**). **f.** CrChR2. Filled circle, a peak component of the photocurrent (I_p). Empty circle, a steady-state component of the photocurrent (I_s). Standard solutions were used (See *Materials and Methods*). Data were presented as the mean ± SEM with *N* = 6 (panel c and f), 7 (panels a, b and e), 9 (panel d).

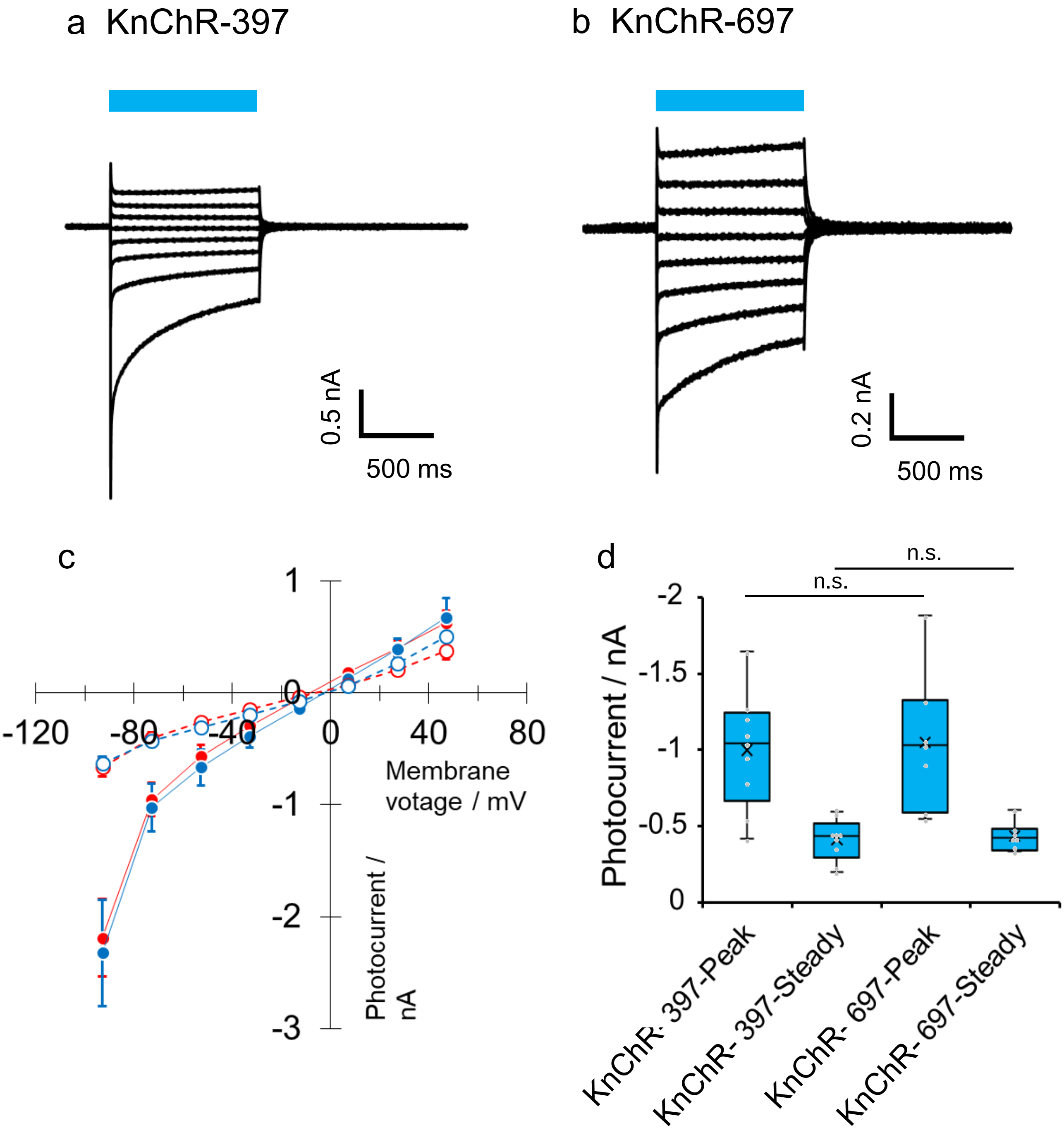

Supplementary Figure 5. Comparison of the length variants KnChR-397 and KnChR-697. Typical photocurrent traces of KnChR-397 amino acids (**a**) and KnChR-697 amino acids (**b**). Blue bars indicate light application (480 nm, 12.3 mW/mm^2). Membrane voltage was clamped from at -90 to +50 mV by 20 mV steps. Standard solutions were used (See *Materials and Methods*). **c**, I-V relationship of KnChR-397 (red) and KnChR-697 (blue). Peak component (filled circle) and steady-state component (empty circle) of the photocurrent were depicted. **d**, Photocurrent amplitudes at -70 mV were shown. Data were presented as the mean±SEM with *N*=9 (KnChR-397), and 7 (KnChR-697).

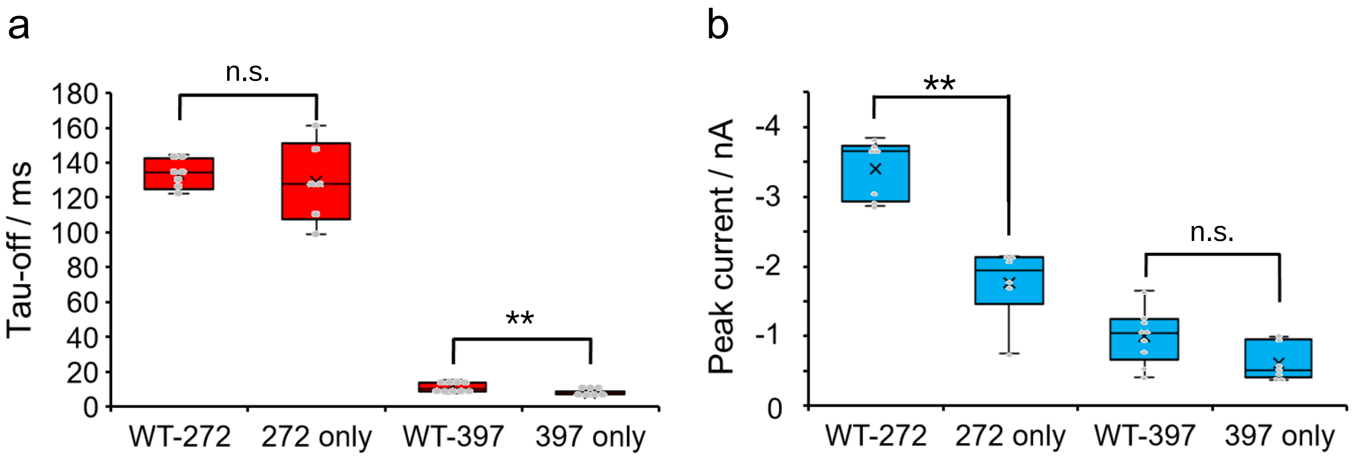

Supplementary Figure 6. eYFP does not alter the channel kinetics of KnChR. Photocurrent amplitudes at -70 mV were analyzed. Time constants (**a**) and peak current amplitude (**b**) of KnChR-272 amino acids and -397 amino acids variants with or without eYFP. Standard solutions were used. Data were presented as the mean ± SEM. *N* =7 (WT-272), 6 (272 only), 9 (WT-397), and 7 (397 only).

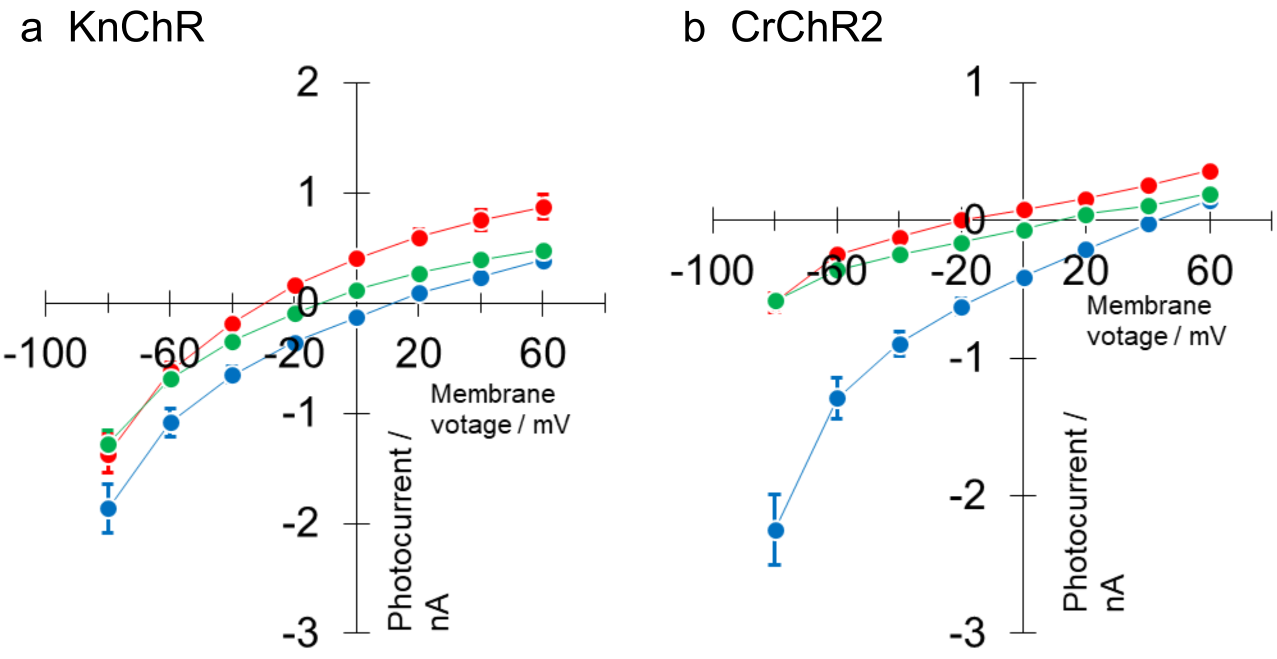

Supplementary Figure 7. I-V relationship of KnChR (a) and CrChR2 (b) in various bath solutions. Red: NMG pH9.0, Blue: K^+ pH9.0, Green: Mg^{2+} pH9.0. See Supplementary Table 4 for more details of solutions. Data were presented as the mean ± SEM. N = 5 (all the conditions for KnChR). N = 8 (CrChR2 in NMG pH 9.0), 5 (CrChR2 in K^+ pH 9.0), and 6 (CrChR2 in Mg^{2+} pH 9.0).

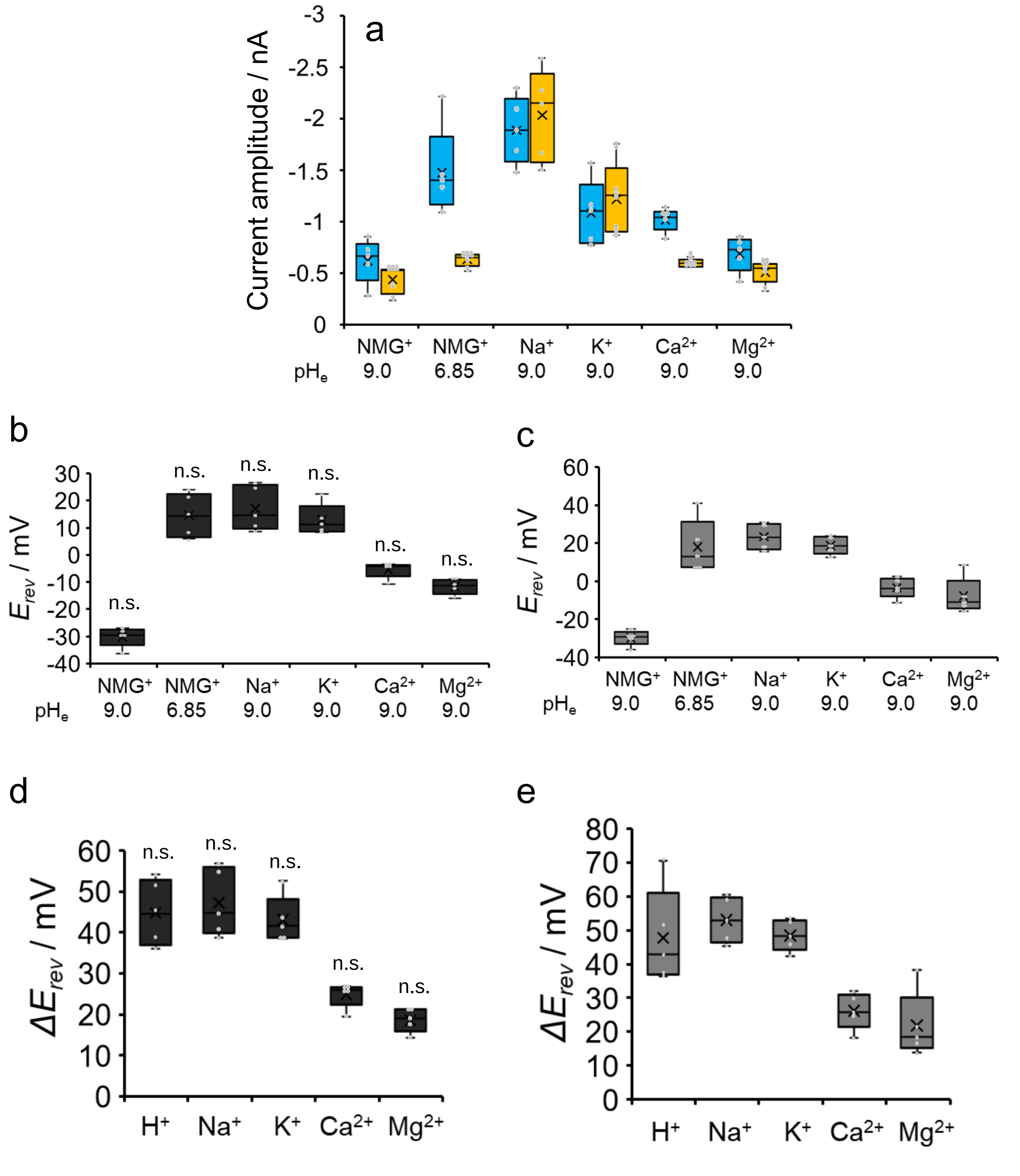

Supplementary Figure 8. Ion selectivity of KnChR. Peak and steady-state component of photocurrents were analyzed. **a**, Photocurrent amplitude at -60 mV under various ionic conditions. blue, peak component. orange, steady-state component. Reversal potential (E_{rev}) of peak component (**b**) and steady-state component (**c**). Shift of E_{rev}, ΔE_{rev} analyzed from peak component (**d**) and from steady-state component (**e**). The Mann-Whitney *U* test was performed between b and c, and d and e. No significant difference was observed between b and c, and d and e respectively. Data were presented as the mean ± SEM with N = 5.

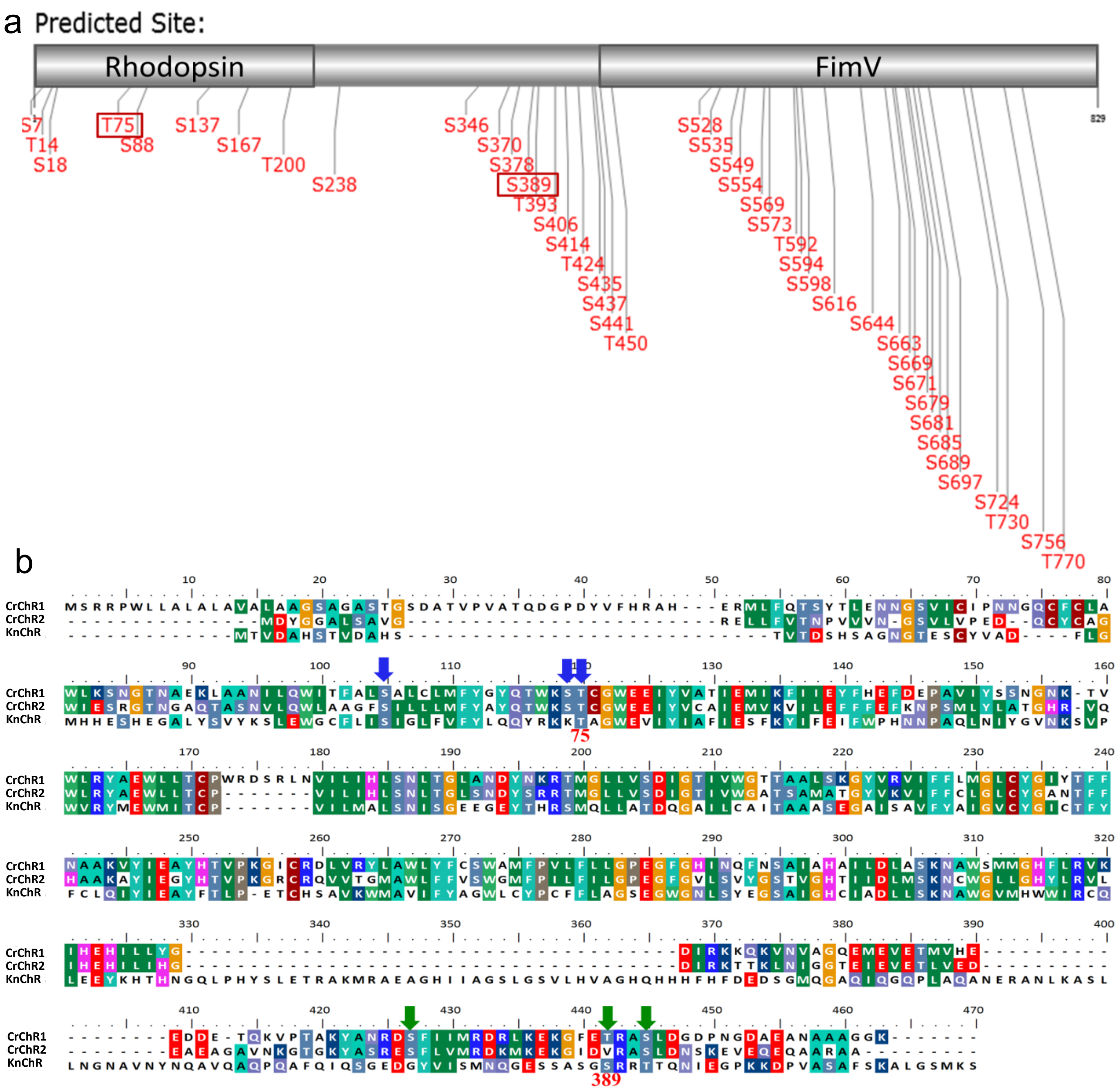

Supplementary Figure 9. Prediction of phosphorylation site in KnChR (**a**) Prediction of kinase-specific phosphorylation site by GPS 5.0 database. T75 and S389 highlighted in red box correspond to Ser102 and Thr373 in CrChR1 phosphorylation site proposed by Böhm et al. (2019) and Wagner et al. (2008), respectively. (**b**) Proposed phosphorylation sites in CrChR1; Böhm et al. (2019) suggested multiple phosphorylation sites, one of them (Ser102) being in the first transmembrane domain (TM) and two (Ser 116 and Thr117) in the intracellular vestibule between (TM) domains marked by a blue arrow. One of them (Thr117) was conserved in KnChR (T75 marked by a red number below the sequence). Wagner et al. (2008) proposed three phosphorylation sites (Ser358, Thr373 and Ser376) in CrChR1 and one in CrChR2 (Ser358) marked by a green arrow. Out of three phosphorylation sites, one is common among CrChR1 and CrChR2 (RD/ESpF) but replaced by Gly in KnChR. Ser389 corresponding to Thr373 is marked by a red number (389) below the sequence.

Supplementary Table 1 Solutions for the ion selectivity measurement

		NaCl	KCl	$CaCl_2$	$MgCl_2$	NMG	EGTA	CHES	MES
**Pipette**	NMG pH9.0	1	1	2	2	140	10	10	-
**Bath**	NMG pH9.0	1	1	2	2	140	-	10	-
	NMG pH6.85	1	1	2	2	140	-	-	10
	Na^+ pH9.0	140	1	2	2	-	-	10	-
	K^+ pH9.0	1	140	2	2	-	-	10	-
	Ca^{2+} pH9.0	1	1	70	2	-	-	10	-
	Mg^{2+} pH9.0	1	1	2	70	-	-	10	-

Supplementary Table 2 List of primers used for insertions

Gene	Sense primers	Anti-sense primers
KnChR_cMyc	TCAGAAGAGGATCTGGGGGATCCAAAGAGC	GATGAGTTTTTGTTCGCTGGCGCTCACAGG
KnChR_272_stop	GTTGGAAGAATACAAGTAAGGGGATCCAAAGAGC	GCTCTTTGGATCCCCTTACTTGTATTCTTCCAAC
KnChR_397_stop	CAACACAGAACATCGAGTAAGGGGATCCAAAGAGC	GCTCTTTGGATCCCCTTACTCGATGTTCTGTGTTG

Supplementary Table 3 List of primers used for subcloning into an eYFP-3.0 vector

Gene	Sense primers	Anti-sense primers
KnChR_272 aa	TTCGAATTCGCCACCGCTAGC ATTATGACAGTGGATG	GCTCTTTGGATCCCCCTTGTA TTCTTCCAACTGGC
KnChR_280 aa	TTCGAATTCGCCACCGCTAGC ATTATGACAGTGGATG	GCTCTTTGGATCCCCTGGCAA CTGTCCGTTATGAG
KnChR_290 aa	TTCGAATTCGCCACCGCTAGC ATTATGACAGTGGATG	GCTCTTTGGATCCCCCATTTT GGCTCTTGTCTCAAG
KnChR_300 aa	TTCGAATTCGCCACCGCTAGC ATTATGACAGTGGATG	GCTCTTTGGATCCCCGCCAGC AATAATATGCCCAG
KnChR_310 aa	TTCGAATTCGCCACCGCTAGC ATTATGACAGTGGATG	GCTCTTTGGATCCCCGCCAGC CACGTGCAAGACGC
KnChR_317 aa	TTCGAATTCGCCACCGCTAGC ATTATGACAGTGGATG	GCTCTTTGGATCCCCGTGAAA ATGATGATGCTGATG
KnChR_321 aa	TTCGAATTCGCCACCGCTAGC ATTATGACAGTGGATG	GCTCTTTGGATCCCCATCCTC ATCAAAGTGAAAATG
KnChR_397 aa	TTCGAATTCGCCACCGCTAGC ATTATGACAGTGGATG	GCTCTTTGGATCCCCCTCGAT GTTCTGTGTTGTCC
KnChR_697 aa	TTCGAATTCGCCACCGCTAGC ATTATGACAGTGGATG	GCTCTTTGGATCCCCGCTGGC GCTCACAGGGGCCC
eYFP 3.0 vector	GGGGATCCAAAGAGCAGGAT	GGTGGCGAATTCGAAGCTTG

Supplementary Table 4 List of primers used for site-directed mutagenesis

Mutations	Sense primers	Anti-sense primers
KnChR_397_E285A	GCCACACTATAGCCTTGCGACAAGAGCCAAAATGA	TCATTTTGGCTCTTGTCGCAAGGCTATAGTGTGGC
KnChR_397_R287A	GTTGCCACACTATAGCCTTGAGACAGCAGCCAAAATGAGG	CCTCATTTTGGCTGCTGTCTCAAGGCTATAGTGTGGCAAC
KnChR_397_K289A	GCCTTGAGACAAGAGCCGCAATGAGGGCTGAAGCTG	CAGCTTCAGCCCTCATTGCGGCTCTTGTCTCAAGGC
KnChR_397_R291A	GAGACAAGAGCCAAAATGGCGGCTGAAGCTGGGCATA	TATGCCCAGCTTCAGCCGCCATTTTGGCTCTTGTCTC
KnChR_397_E293A	AGCCAAAATGAGGGCTGCAGCTGGGCATATTATTG	CAATAATATGCCCAGCTGCAGCCCTCATTTTGGCT
